# Incorporating Torsion Into 3D Geometric Reconstruction of Left‐Ventricle From 2D Echocardiography and Its Impact on Hemodynamics

**DOI:** 10.1002/cnm.70220

**Published:** 2026-10-08

**Authors:** Sai Sree Chandra Sirani, Hossein Geshani, Minako Katayama, Syed Samar Abbas, Marek Belohlavek, Iman Borazjani

**Affiliations:** ^1^ J. Mike Walker '66 Department of Mechanical Engineering Texas A&M University College Station Texas USA; ^2^ Department of Cardiovascular Medicine Mayo Clinic Scottsdale Arizona USA

**Keywords:** computational fluid dynamics, echocardiography, hemodynamics, immersed boundary method, left ventricular torsion

## Abstract

In addition to systolic contraction and diastolic relaxation, torsional motion is an essential component of left ventricle (LV) wall motion, but its effect on LV hemodynamics is not fully understood. To this end, the measured rotation of short‐axis sections is incorporated into our 3D reconstruction of LV from 2D echocardiography, which serves as input to the curvilinear immersed boundary framework for LV flow simulations. The geometry is validated against sonomicrometry measurements, whose difference is found to be within the typical myocardium thickness range, and the flow is validated against intracardiac flow velocity measured by Doppler ultrasound. To assess the impact of torsion, two simulations (one including torsion and one without in the reconstruction from the same set of images) are compared. It is found that including torsion in reconstruction for a normal LV does not change the geometry, and consequently the volume and flow rates, due to the circular shape of LV short‐axis sections. However, the time‐averaged wall shear force increases by 12.6% during systole and drops by 4.9% during diastole when torsion is included. Torsion also reduces washout efficiency for this subject, as the volume of fluid with residence time under one cycle is 16.6% lower, while fluid staying 1–2 cycles increases by 3.7%. Viscous frictional energy loss decreases by 3.8% for LV with torsion.

## Graphical abstract

Torsion is incorporated into our 3D reconstruction method from 2D echocardiography for flow simulations. The simulations are validated against our measurements. With the inclusion of torsion in reconstruction, the bulk parameters remain the same but time‐averaged wall shear stress increases or decreases during systole or diastole, respectively, while residence time in the left ventricle increases.

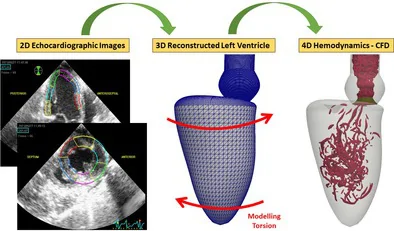

## 1 Introduction

The left ventricle (LV) function is crucial as it systematically circulates the oxygenated blood to the rest of the body. Deviation in LV mechanics can lead to different cardiac disorders and heart failure [1, 2, 3, 4]. 2D Echocardiography (echo) is the most widely used imaging modality due to its low cost, fast acquisition time, and wide availability for noninvasive assessment of LV function and its abnormalities through commonly used parameters such as shape, size, ejection fraction (EF), and strain [5]. Nevertheless, 2D echo (aside from Doppler measurements) and most other imaging modalities (apart from 4D‐flow magnetic resonance imaging, MRI) cannot capture the LV's full 4D hemodynamics. Image‐based computational fluid dynamics (CFD) simulations [6, 7, 8, 9, 10, 11] have been routinely used to obtain LV hemodynamics for research. However, clinical use of image‐based CFD has been limited due to many challenges such as limited availability of images, low temporal–spatial resolution of images, and time‐consuming computations. Combining 3D LV reconstruction based on 2D echo with CFD^12^ integrates the benefits of 2D echo (such as wider clinical availability and high temporal resolution) with CFD to overcome some of the challenges and obtain 4D flow.

Torsion is an essential component in LV mechanics. Apart from LV deformation, which includes longitudinal, circumferential, and radial strains [12], LV also undergoes torsion. The LV torsion is often referred to as wringing motion and is characterized by systolic twisting and diastolic untwisting [13]. Due to the helical arrangement, the orientation of myocardial fibers of the LV changes from a left‐handed helix in the subepicardium to a right‐handed helix in the subendocardium [14]. This helical pattern enables LV to undergo torsion in which the apical short‐axis (SA) section rotates counterclockwise and the basal SA section rotates clockwise during systole when viewed from the apex. The helical pattern of the fibers and the resulting torsional motion is believed to enable the heart to achieve an EF of 60%–70%. Without torsion, relying solely on myocardial contraction, EF would be limited to 15%–20% [15]. LV twist is the net rotation between apical and basal sections, whereas torsion is defined as a 2D in‐plane shear angle computed from the corresponding 2D rotations [16, 17, 18].

Since torsion is an integral aspect of LV mechanics, understanding its impact on 4D flow behavior is of particular interest. Nevertheless, in many image‐based CFD studies, torsional motion compared to radial and axial motions of the LV wall has sometimes been ignored [6, 7, 8, 9, 19]. In these image‐based simulations, in contrast to structural [17, 20] and fluid–structure interaction simulations [21], the torsional motion does not affect the ejection fraction (LV volume) because LV volume is obtained from images of a heart that has torsion and only ignored in the LV reconstruction from images. In a cine MRI‐based study by Canè et al. [10], it was reported that including torsion in the reconstructed LV from the same set of images reduces LV washout, which is reflected in the increased residence time within the LV. Another MRI‐CFD study [11] also reported that torsional motion, combined with internal LV structures, contributes to blood stagnation. This conclusion was drawn from variations in the relative residence time (RRT) parameter across models with different maximum torsion angles. Apart from the above two studies, we found no other studies that directly investigate the effects of torsion on LV hemodynamics. Indeed, it is not clear how torsion affects the wall shear stress, energy loss, and general flow features of the flow. To answer these questions, the torsional motion is incorporated into the 3D reconstruction from 2D echo (Section 2.2). The 3D LV geometry with and without torsion, reconstructed from the same set of echo images of an animal with normal torsional motion, is placed as input to our in‐house CFD code that can handle large deformation of wall boundaries using a sharp‐interface immersed boundary method (Section 2.3). The geometry and flow are validated against measurements, and the effects of torsion are discussed in Section 3. The results are compared to earlier work in Section 4, and the main findings are presented in Section 5.

## 2 Methodology

### 2.1 2D Echo Dataset Acquisition

The study was approved by the Institutional Animal Care and Use Committee. Adult domestic pigs weighing approximately 58kg were used. Each animal was fasted overnight, sedated prior to the procedure, intubated, fully anesthetized, and mechanically ventilated. A medial sternotomy was performed to open the chest, and the heart was suspended on a pericardial cradle to provide better access to the left ventricle (LV) surface for ultrasound imaging. In this open‐chest setup, the ultrasound transducer was placed directly on the heart, with a layer of ultrasonic gel applied to ensure proper acoustic coupling. 2D short‐axis (SA) projections of the LV were acquired using a Vivid 7 or E95 ultrasonography system (GE Healthcare) equipped with a phased‐array transducer (M4S or M5Sc‐D). The grayscale video clips were digitally recorded and transferred to EchoPAC software v206 (GE Healthcare) for analysis. Speckle‐tracking algorithms were employed by EchoPAC to detect and track myocardial rotational motion throughout the cardiac cycle. The time histories of both basal and apical rotations, where the angular data (in degrees) were sampled at a temporal resolution of 18 ms, were used to construct the angle versus time curves shown in Figure 1a. These were subsequently combined to produce a twist curve representing the net LV torsional motion (Figure 1a).

**FIGURE 1:**
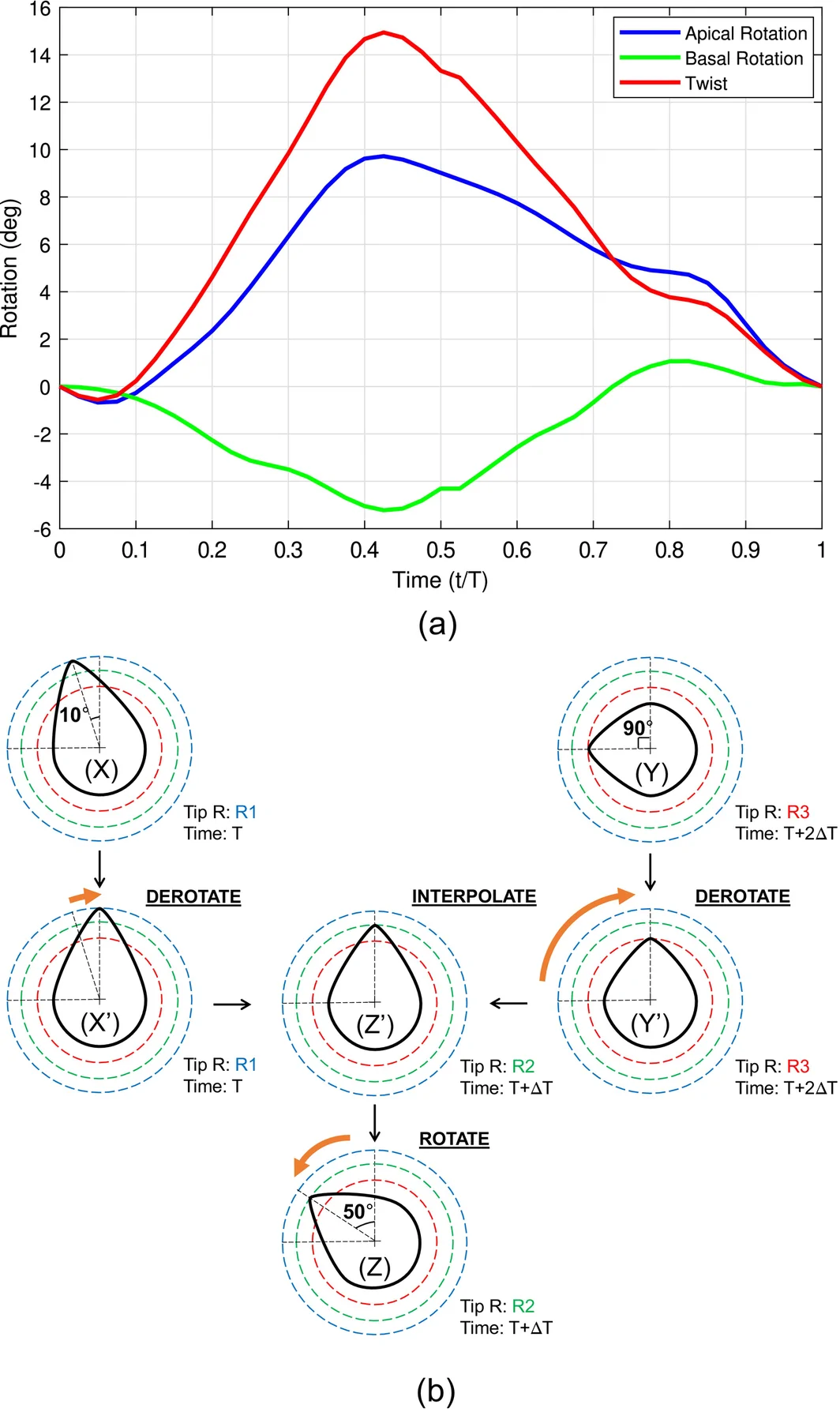
(a) Time history of basal and apical rotations along with computed twist over a cardiac cycle. (b) Temporal interpolation of intermediate shape (Z) capturing deformation and rotation of a simplified basal SA section.

Sonomicrometry (sono) is used to verify the 3D reconstructed LV geometry. Sono measures instantaneous myocardial thickness or LV dimensions [22]. Round transducers (piezoelectric crystals about 2 mm in diameter) are sutured epicardially or implanted endocardially around the LV. The distance between any two crystals is calculated based on a time of flight of a 1‐MHz ultrasonic pulse between them. Using a triangulation algorithm, an array of displacement measurements is converted into a system of 3D coordinates [23].

Doppler velocity assessment is a noninvasive ultrasound technique used to display blood flow and measure its velocity in cardiovascular systems. Pulsed wave (PW) Doppler provides a spectrum from a user‐defined sample volume location within the heart or vessel. PW Doppler echo techniques are essentially based on a shift in Doppler pulse frequency induced in a signal reflected from moving red blood cells [24]. Here, the PW Doppler method is applied to beating hearts of anesthetized pigs to record the time history of local blood velocities.

### 2.2 LV 3D Reconstruction From 2D Echo

The 3D LV geometry is reconstructed from 2D echo by incorporating torsion into an algorithm described in our previous studies by Krishnan et al. [25] and Hedayat et al. [19]. To provide an overview, the algorithm [19] involves identifying the endocardial outlines of LV in six standard long‐axis (LA) and SA echo projections. Apical three (A3C), two (A2C), and four (A4C) chamber views of the LV are considered LA projections, while basal, mid, and apical views are SA projections. The outlines are smoothened and temporally interpolated using B‐spline and GNU Scientific Library (GSL) cubic spline methods respectively. Next, the processed LV outlines are stitched together and, based on a bivariate type spatial interpolation, all the LV surface points are generated. Finally, by connecting these points using a triangular mesh and temporally smoothening the surface, the 3D reconstructed LV geometry is obtained.

The torsional motion of the LV is quantified through SA sections of 2D echo as discussed in Section 2.1 and shown in Figure 1a. To model torsion, the existing reconstruction algorithm [19] is altered during temporal interpolation stage for SA sections. To illustrate the interpolation process, consider a simplified basal section in Figure 1b, where the shape transitions from (*X*) to (*Y*), and the objective is to accurately reconstruct the intermediate shape. This procedure is equally applicable to the apical and mid sections. Deformation is represented by a reduction in the projected tip radius, from R1 at time *T* (blue circle) to R3 at time T+2ΔT (red circle). Rotation is indicated by a change in the angular orientation of the tip, from 10° to an exaggerated 90° with respect to the vertical, to clearly visualize the rotational component. The desired shape, labeled (*Z*), is the result of an counterclockwise rotation of 50° (linear interpolation between 10° and 90°) combined with a reduction in radius from R1 to R2 (green circle).

The first step is to reorient the tips of both configurations (*X*) and (*Y*) to a reference vertical angle of 0°. This removes the rotational component and retains only the deformation, as seen in shapes (*X*') and (*Y*′). The de‐rotated shapes are then temporally interpolated where the radius reduces to R2. Following this, the tip is rotated anticlockwise by 50° to achieve the desired orientation. The same process is applied to all SA sections from base to apex. The rotation angle for any section is the linear interpolation between basal and apical rotation.

### 2.3 Hybrid Echo‐CFD Method for Flow Simulations

The ventricular flow is simulated by the motion of the 3D reconstructed LV system using a sharp‐interface curvilinear immersed boundary (CurvIB) flow solver [26, 27, 28]. This in‐house CFD code has been extensively validated for problems involving complex flow fields [29, 30, 31] and applied in various domains: cardiovascular flows [6, 32, 33, 34, 35, 36, 37]; vortex‐induced vibrations [38, 39]; and aquatic fish locomotion [40, 41, 42, 43], low Reynolds and rheology flows [44, 45, 46] and turbulent flows [47, 48, 49, 50].

In this method, the immersed LV geometry, meshed into triangular elements, is embedded at each time‐step within a curvilinear background grid (Figure 2a). Based on the motion of the LV boundaries, grid nodes are classified into fluid, immersed boundary, and solid nodes using an efficient ray‐tracing algorithm [28]. Heart valves are modeled as thin structures, and their immersed boundary nodes are handled using a specialized algorithm designed for thin bodies [51].

**FIGURE 2:**
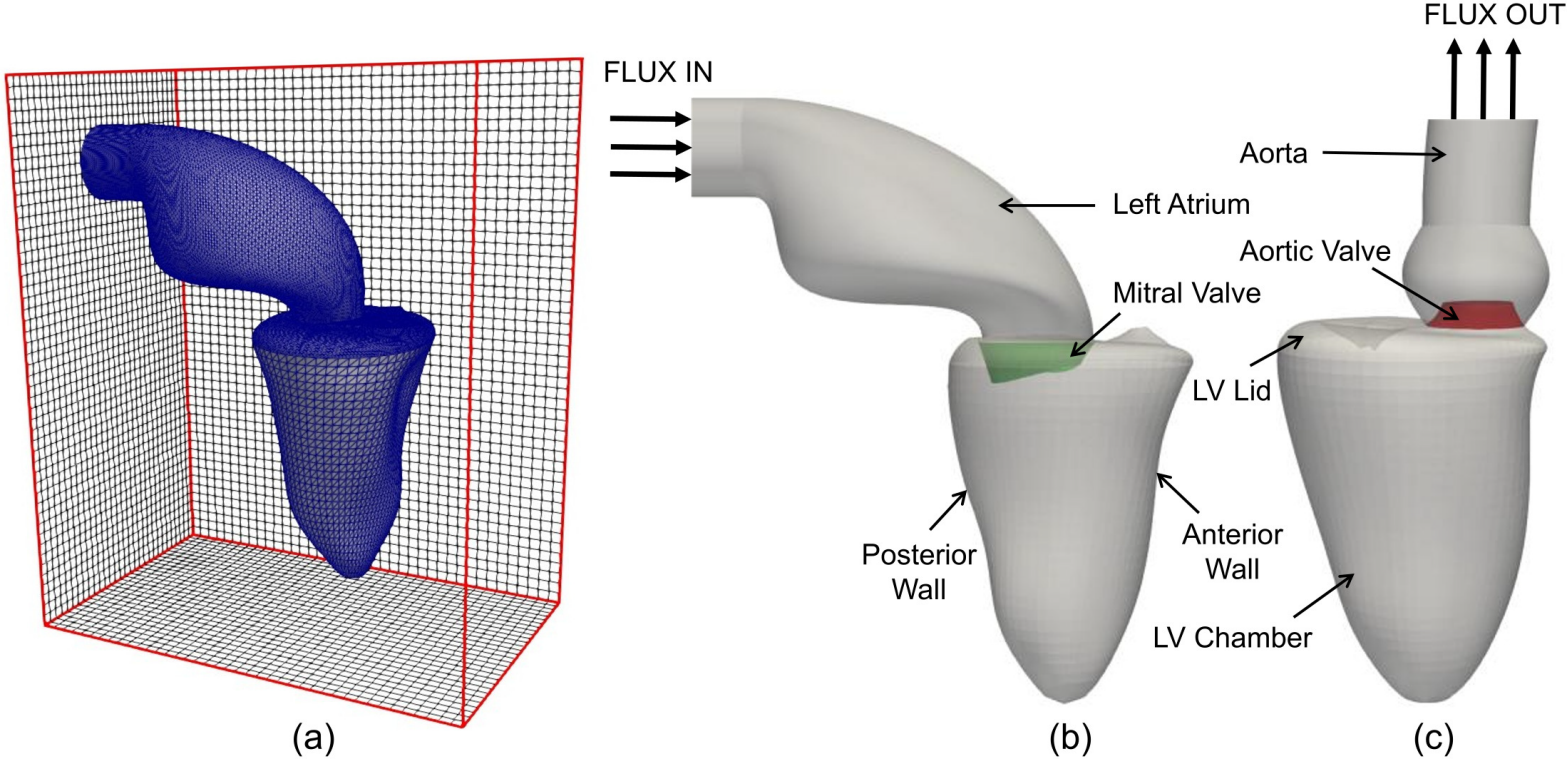
(a) LV system during diastole with triangulated surface mesh immersed in a background cartesian grid. Assembly of the approximated geometries (aorta, left atrium, and LV lid) with the 3D reconstructed LV chamber during (b) diastole and (c) systole phases, respectively.

After node classification, the 3D unsteady incompressible continuity and Navier–Stokes equations are solved over the fluid region using a fractional step method [27]. In the fractional step method, the momentum equations are solved implicitly [27, 31] while the Poisson equation is solved using Generalized Minimal Residual (GMRES) method with multigrid as a preconditioner. A no‐slip boundary condition is imposed at the fluid–solid interface based on the velocity of the immersed LV surface. Blood flowing into and out of the LV chamber is governed by the conservation of mass, that is, the net inflow or outflow (volume flux) equals the rate of change in LV volume between two consecutive time steps. For diastole, the LV chamber, mitral valve, and atrium are immersed together, and the volumetric change is applied as an inflow flux at the atrium (Figure 2b). For systole, as shown in Figure 2c, the immersed geometry includes the LV chamber, aortic valve, and aorta, with the volumetric change applied as an outflow flux at the aorta. Our model assumes that at any given time instant, only one valve (either mitral or aortic) is open, while the other remains closed and sealed with its leaflets in our setting with healthy experimental pigs.

Two numerical simulations are conducted: one with the LV geometry modeled without torsion (referred to as torsion OFF) and the other with torsion (torsion ON). The cardiac cycle duration, based on the echo images, is T=0.705s and each simulation is ran with a temporal discretization of 2500 time instances (time step size $\Delta t=0.282\times 10^{-3}\;s$). Based on the 3D reconstruction, the aortic diameter is D=19mm, and the bulk velocity (peak systolic flow rate divided by the aortic orifice area) is $U_{\text{bulk}}=0.631\;m/s$, which are used to non‐dimensionalize the governing equations. The blood viscosity is set to $\nu =3.3\times 10^{-6}\;m^{2}/s$ and the Reynolds number is computed as Re=UbulkD/ν=3630. Blood is modeled as a Newtonian fluid, which has been reported to be a reasonable assumption for flow in the heart chambers and large arteries including aorta [36, 52, 53]. Due to transitional and pulsatile nature of LV flows, we chose not to use turbulent models available in our code [47, 48, 49, 50]. Based on our past experience for transitional biomedical flows [6, 19, 36], using turbulence models increases the diffusion and makes the flow more organized. The LV geometry is immersed in a background grid of size 5.19D×3.33D×6.32D, consistent with prior work [19], and discretized using 241×181×301 grid points along the respective Cartesian directions. This grid resolution is selected based on a grid sensitivity study (Section 3.2). Results from the second cardiac cycle, which demonstrate small cycle‐to‐cycle sensitivity afterwards (Section 3.2), are used for analysis. These grid and cycle independent studies are shown in the results Section 3.2.

### 2.4 Flow Field Analysis

LV performance is evaluated based on the viscous frictional energy loss $\left(e_{\text{loss}}\right)$. Using the conservation of energy equation derived from the Reynolds Transport Theorem (RTT), the energy loss within a control volume (i.e., the LV) is expressed as follows:

(1) $$\frac{{\mathrm{dE}}_{\text{loss}}}{\mathrm{dt}}=\dot{Q}-\dot{W}=\frac{\partial }{\partial t}\int_{\mathrm{CV}}\rho e\;\mathrm{dV}+\int_{\mathrm{CS}}\left(\rho e+p\right)\;\vec{U}\cdot d\vec{A}$$

(2) $$e=u_{\mathrm{int}}+\frac{U^{2}}{2}+\mathrm{gz}$$

where $E_{\text{loss}}$ is the total system energy, $\dot{Q}$ is the heat transfer rate to the system, $\dot{W}$ is the rate of work done by/on the system, CV and CS are the control volume and surface respectively, ρ is the density of blood, p is pressure, $\vec{U}$ is the flow velocity, dV is differential volume element, $d\vec{A}$ is differential outward area vector, t is time and e is the energy of the fluid per unit mass which is the addition of $u_{\mathrm{int}}$ (internal energy), $\frac{U^{2}}{2}$ (kinetic energy) and gz (potential energy). By neglecting heat transfer to LV, internal energy and gravity, the time integrated energy equation over a cardiac cycle reduces to,

(3) $$E_{\text{loss}}=\int_{\mathrm{CV}}\rho \left(\frac{U_{T}^{2}}{2}-\frac{U_{0}^{2}}{2}\right)\;\mathrm{dV}+\int_{0}^{T}\int_{\mathrm{CS}}\left(\frac{\rho U^{2}}{2}+p\right)\vec{U}\cdot \vec{d}A\;\mathrm{dt}$$

(4) $$e_{\text{loss}}\left(J/\mathrm{lit}\right)=\frac{E_{\text{loss}}}{\mathrm{SV}}$$

where T is the cycle end time. $e_{\text{loss}}$ calculates the viscous energy loss per liter of blood pumped.

The residence time ($T_{R}$) quantifies the duration for which fluid particles stay inside the domain of interest. It increases with time t as a fluid particle enters a domain at initial time $t_{0}$, that is, $T_{R}=t-t_{0}$. The time derivative following a particle, therefore, is ${\mathrm{DT}}_{R}/\mathrm{Dt}$ = 1, and using the definition of the material derivative results in the convection equation with a unit source term as follows [37]:

(5) $$\frac{{\mathrm{DT}}_{R}}{\mathrm{Dt}}=\frac{\partial T_{R}}{\partial t}+\nabla \cdot \left(uT_{R}\right)=1$$

A Dirichlet boundary condition of $T_{R}=0$ is applied at the atrial inlet, while a Neumann boundary condition ($\partial T_{R}/\partial n=0$) is imposed at the aortic outlet and LV walls in the normal direction. The Neumann outflow condition forces the fluid (and $T_{R}$) to exit smoothly without accumulating or reflecting at the boundary. $T_{R}$ is an indicator of the degree of flow stasis/stagnation (and simultaneously the washout) and will be investigated in this work.

## 3 Results

### 3.1 LV Reconstruction Comparison Against Sonomicrometry Data

Figure 3a shows the 10 sonomicrometry (sono) crystal locations along with the 3D reconstructed left ventricle (LV) for the baseline condition. The vertices of the triangles indicate the positions of the crystals. Crystal C10 served as an external spatial reference for Cartesian coordinates. Crystals C1‐C3 were sutured to the LV epicardial surface to protect the mitral valve leaflets, fibrous cords, big coronary branches, and other adjacent structures. Crystals C4‐C9 were inserted into the subendocardial layer under ultrasound guidance. The crystals determine heart dimensions with sub‐mm accuracy at a rate of approximately 250 Hz (practical experience). However, some spatial discrepancies between the sono crystal placements and the 3D reconstructed LV surface could have been caused by sono limitations. Crystals C1‐C3 measured mutual epicardial distances, whereas the 3D reconstruction represents the endocardial surface. Crystals C4‐C9 tracked (a) myocardial spiral twisting, which was minimal at the mid‐level (which in part explains the least error at this level) but particularly strong at the apical level, and (b) LV shortening along its longitudinal axis, both phenomena resulting in variable out‐of‐plane movement on short‐axis (SA) ultrasound imaging. However, motion tracking could have been unpredictably impaired due to (i) the squeezing effect on the crystals by the surrounding contracting muscle and (ii) the pull on the crystals by their connecting wire. For the full cardiac cycle, Figure 3a presents the error plot between the mid‐plane crystals and the LV surface. The error is computed as the shortest distance from each crystal to the reconstructed surface at any given time instance. Among all the crystals, crystal 5 exhibits the highest error, with values of 3.36mm at end systole (ES) and 5.37mm at end diastole (ED). It also records an overall maximum error of 5.62mm across the entire cardiac cycle. These values are considered acceptable, as they remain below the minimum (6mm) and maximum (12mm) surface distance errors reported at mid segments when comparing 3D echo and cardiac MRI geometries [54]. Additionally, the echo–sono errors in the current study fall within a justifiable range when compared to posterior wall thickness, which varies along the mid‐LV region from 7.7±1.7mm (ES) to 5.1±0.8mm (ED) as measured by cardiac MRI [55].

**FIGURE 3:**
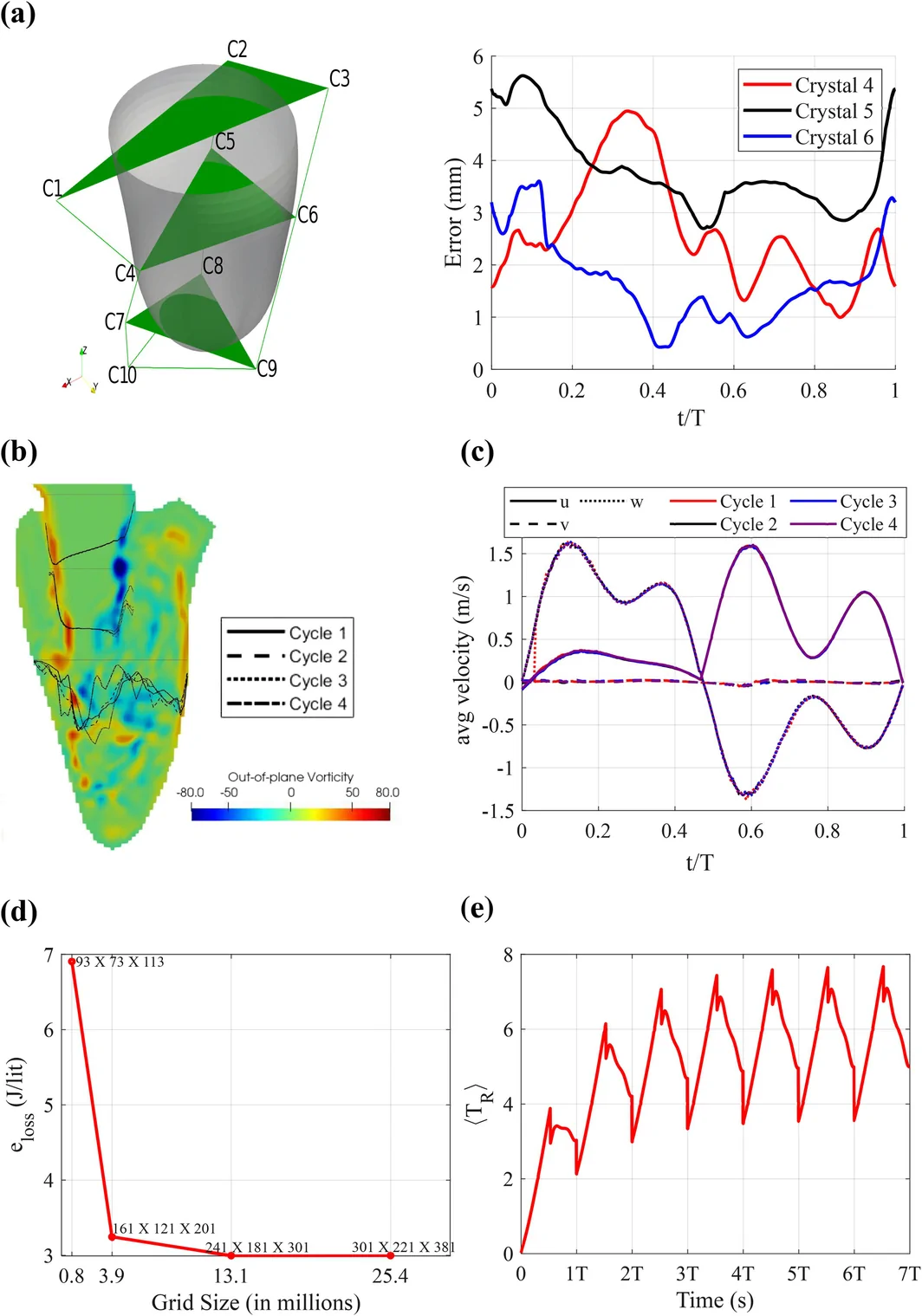
(a) Comparison of sono crystals placement with 3D reconstructed LV surface at early systole along with time history of shortest distances (error) computed between mid SA crystals and LV surface. (b) Peak diastole cycle‐to‐cycle variation of velocity line plots at different locations on a background vorticity contour. (c) Cycle‐to‐cycle comparison of volume‐averaged mean velocity components. (d) $e_{\text{loss}}$ versus grid size plots for torsion OFF configuration. (e) Time history of volume‐averaged residence time across cardiac cycles.

### 3.2 Cardiac Cycle‐To‐Cycle Variation Analysis and Grid Sensitivity Studies

The cardiac cycle‐to‐cycle variation analysis and grid sensitivity studies are conducted on the torsion OFF configuration. First, the number of cycles required to achieve convergence is determined using a grid size of 161×121×201. Figure 3b presents a cycle‐to‐cycle comparison of three velocity line plots at different locations during peak diastole. It is evident that the variation becomes negligible beyond cycle 2 near the mitral valve. However, further towards the apex of the LV chamber, more noticeable variation is observed, as indicated by the line plot at the mid SA location. These cycle‐to‐cycle variations are due to translational nature of the flow in the mid‐apical region as shown by the presence of multiple smaller eddies in the mid‐apical region in the background mid‐plane vorticity contour (Figure 3b). Nevertheless, the velocity fluctuations below the midplane beyond the first cycle are in the same order which justifies using the second cycle. Indeed, the averaged quantities remain similar from the second cycle onward as shown in Figure 3c by the spatially averaged velocity plots throughout the cycle. The volume‐averaged kinetic energy and mean velocity components show cycle‐to‐cycle differences below 3.5% and 2.7%, respectively, from the second cycle onward, confirming that the flow is periodic in an average sense despite point‐wise variation.

For cycle‐to‐cycle sensitivity of $T_{R}$, $T_{R}$ is computed using the flow field from the second cycle, for several cardiac cycles. The cycle‐to‐cycle change in volume‐averaged $T_{R}$, plotted in Figure 3e, decreases monotonically from cycle to cycle. After the seventh cycle, the volume‐averaged $T_{R}$ value differs by less than 0.22% from the sixth cycle, indicating that the solution has reached a periodic state and is cycle‐to‐cycle independent. A very similar trend and convergence behavior is observed for the torsion OFF case, although only the torsion ON case is plotted in Figure 3e. Therefore, the results from the seventh cycle were used in the analysis.

For grid sensitivity, $e_{\text{loss}}$ values from the second cardiac cycle are compared across simulations performed with varying grid sizes: the original size (×0.5), (×1.5), and (×2.0). As shown in Figure 3d, the $e_{\text{loss}}$ for the 25.4 M grid differs by less than 0.05% compared to the 13.1 M grid, where M denotes million. Based on this observation, all simulations are carried out for 2 cardiac cycles using a fixed grid resolution of 241×181×301.

### 3.3 Validation of Simulated Flows Against Doppler Measurements

For validation, the simulated flow field is compared against available PW Doppler measurements at aortic and mitral orifices during systole and diastole respectively. Before validation, a plane sensitivity analysis is first performed to ensure that the computed area‐averaged velocity does not depend on the chosen area. Figure 4c shows a horizontal plane positioned along the sinus of Valsalva, close to the experimental plane for PW Doppler, with two additional planes located above and below it, separated by a distance of 0.05D. The area‐averaged $U_{\mathrm{mag}}$ recorded during systole shows no significant variation across these planes. This analysis confirms that the results are independent of the chosen sampling plane in the numerical simulation and should corresponds well to the location of the sample volume in PW Doppler.

**FIGURE 4:**
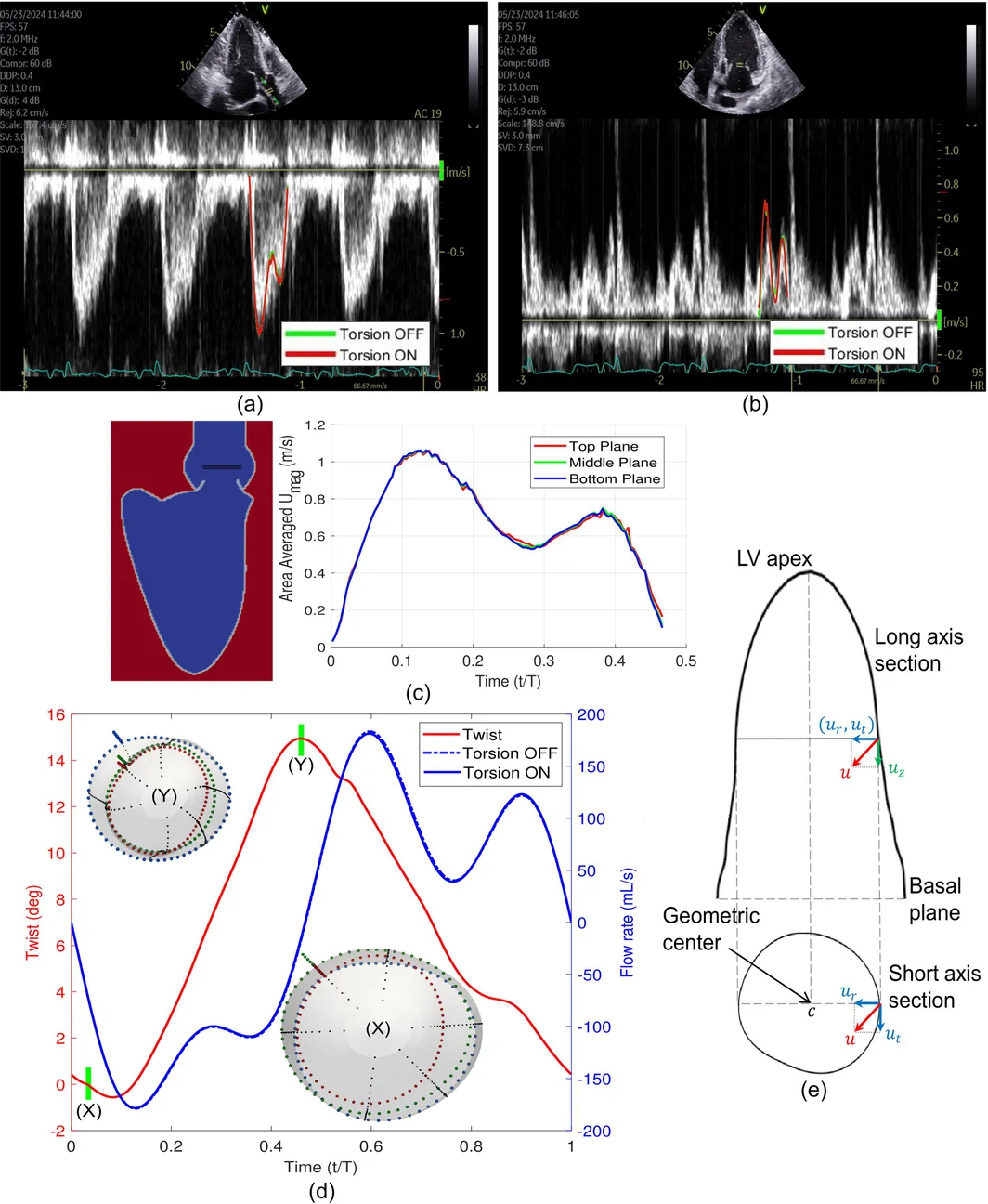
Comparison of Doppler velocity measurements with the velocity magnitude from numerical simulation (a) at aortic orifice during systole and (b) at mitral orifice during diastole. (c) Plane independent study: Time history of area‐averaged velocity on different planes near aortic orifice during systole shows negligible difference. (d) Time history of twist and flow rate. View of LV from the apex at (*X*) zero and (*Y*) peak twist to visualize torsion. (e) Decomposition of the velocity u at a point on the LV surface into its cylindrical, that is, radial $u_{r}$, tangential (torsional) $u_{t}$, and axial $u_{z}$, components.

Figure 4a,b presents comparison between CFD‐simulated velocities and experimental Doppler measurements of intracardiac flow velocities during systole and diastole. The numerical results show good agreement with the Doppler readings. Between torsion OFF and ON cases, the velocity trends almost match with each other. The numerical velocity captures the E‐ and A‐wave during diastole (Figure 4b). Two peaks, smaller than the ones during diastole, are also observed in the velocity profile during systole (Figure 4a). The notch in the simulations seems to be larger than the one in the Doppler measurements (Figure 4a). The double peak during systole is unexpected for healthy hearts and may be due to placing the heart on a pericardial cradle during the open chest measurements, direct contact of the transducer with the heart during the scan, or altered heart motion due to a bulk of acoustic gel placed on the heart to ensure acoustic contact.

### 3.4 The Effects of Torsion

Torsion modeling in the 3D reconstructed LV appears as rotation of SA sections as discussed in Section 2.2. In Figure 4d, systolic twisting is visualized as a change in the orientation of the SA reference markers from (*X*) to (*Y*). This torsional motion is accompanied by the shrinking of the LV cavity. A peak twist of 15° is observed by the end of systole. During diastole, the SA sections undergo untwisting, returning the LV to its relaxed state. Figure 4d also shows the flow rate curves characterized by two systolic peaks and two diastolic peaks, known as the E‐wave and A‐wave. The E‐wave results from rapid early inflow when the pressure difference between the left atrium and LV causes the mitral valve to open, initiating ventricular filling. The A‐wave corresponds to a second filling phase driven by atrial systole.

It remains difficult to visually appreciate torsion when observing the entire LV surface between the torsion ON and OFF cases because the SA slices are approximately circular in shape (mean aspect ratio 1.03–1.10 across basal, mid, and apical sections) whose rotation does not drastically change their shape, as confirmed by the cross‐sectional shape analysis in Figure 5. Furthermore, the twist between two consecutive time instants is small (of the order 0.015°). As a result, the overall geometry of the LV remains unaltered when torsion is included in the reconstruction. The volume of the LV chamber remains nearly identical at each time instance between the torsion OFF and ON cases. Consequently, the flux curve (Figure 4d), bulk velocity and SV (Table 1) show no significant variation due to torsion modeling. The addition of torsion results in a 3.8% reduction in viscous frictional energy loss $\left(e_{\text{loss}}\right)$.

**FIGURE 5:**
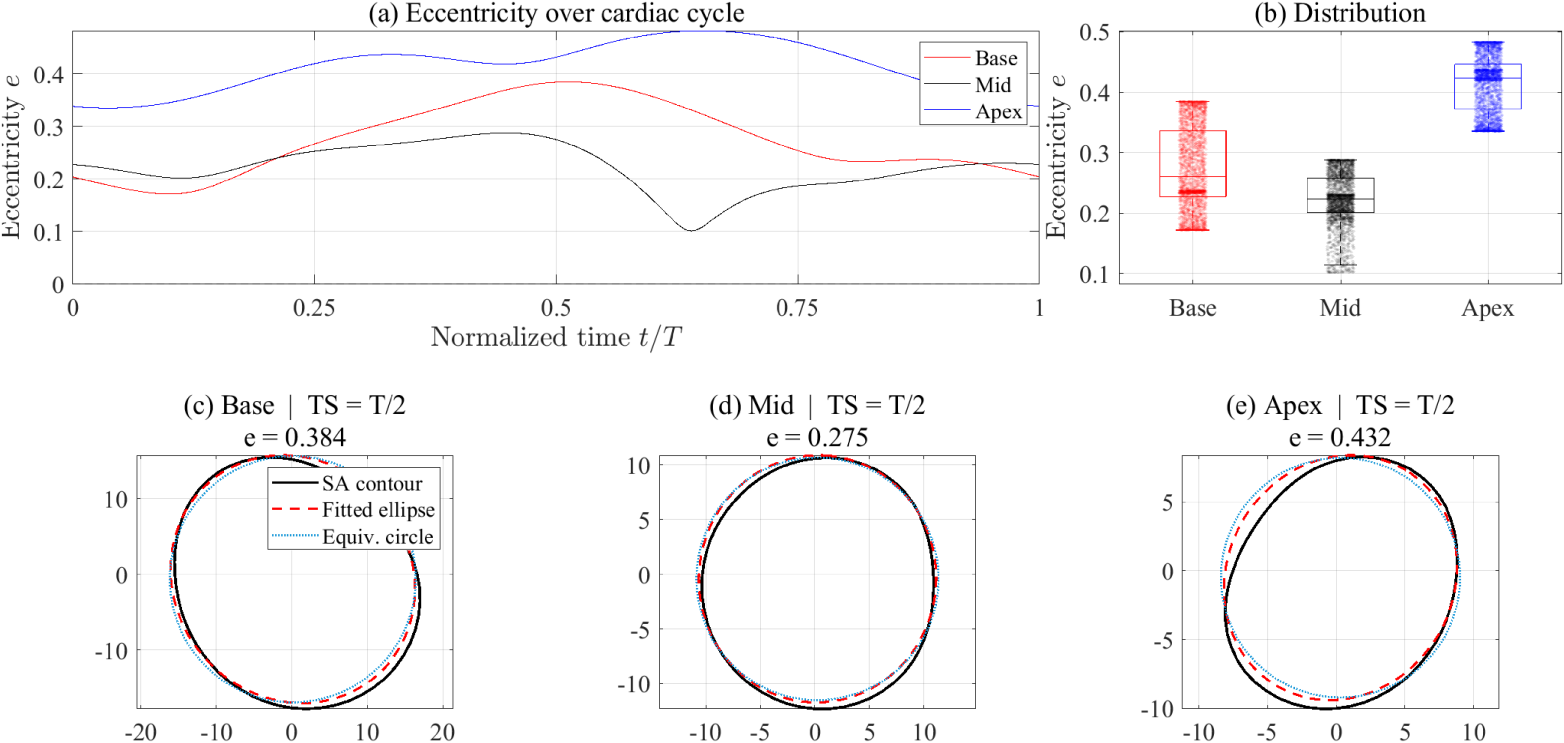
A cross‐section shape analysis. (a, b) Eccentricity time series over the cardiac cycle for the basal, mid‐ventricular, and apical sections. (c–e) Representative basal, mid, and apical cross‐sections, respectively, at t/T=0.5, showing the SA contour (solid), fitted ellipse (red dashed), and area‐equivalent circle (blue dotted).

**TABLE 1:** Comparison of LV cardiac parameters between torsion OFF and ON configurations.

Category	Torsion OFF	Torsion ON
Bulk velocity (m/s)	0.631	0.629
SV (mL)	36.6	36.4
$e_{\text{loss}}$ (J/lit)	2.993	2.879

While LV geometry is not affected by including torsion into the reconstruction from echo, the LV velocity is impacted. At each node on the LV surface, the velocity is decomposed into cylindrical components $\left(u_{r},u_{t},u_{z}\right)$, where $u_{r}$ and $u_{t}$ represent the radial and tangential components, respectively, on the short‐axis (SA) plane, and $u_{z}$ aligns with the LV's longitudinal axis in the z direction, as shown in Figure 4e. The tangential component $u_{t}$ arises due to torsional motion and is zero across all nodes in the torsion OFF case. To quantify the change in surface velocity due to torsion, the ratio of $u_{t}$ to the total velocity magnitude is calculated over LV surface (surface shown in Figure 3a and the basal cap), then spatially (area) averaged. This metric captures the relative contribution of torsion to LV surface motion. Over a single cardiac cycle, the area‐averaged ratio reaches a maximum of 46.91%, a minimum of 2.88%, and its cycle‐averaged value is 20.58%.

The LV surface velocity, affected by torsion, should modify the behavior of fluid adjacent to LV chamber wall and the wall shear stresses more than other regions. To evaluate this effect, wall shear stress (WSS) on the LV surface is analyzed. The nondimensional shear magnitude is defined as the tangential velocity gradient along each element's area‐normal direction. Figure 6 displays the WSS contour, with the LV surface split along the mid‐plane (A3C view). Both cases exhibit high WSS in the mid‐plane region during early systole as a result of A‐wave filling from the previous cycle (Figure 6a). The stresses subsequently decrease as blood continues to empty from the LV (Figure 6b). WSS rises again across the entire LV surface due to the onset of diastolic E‐wave inflow and then gradually declines throughout diastole (Figure 6c,d). This contour pattern repeats in each subsequent cycle. To highlight the difference in WSS due to torsion and to derive a more quantitative understanding, WSS is evaluated over the entire LV surface to compute the LV wall shear force. During systole, the shear force is higher for the torsion ON case, with a systolic phase time‐averaged value of 0.0206, which is 12.56% greater than that of torsion OFF. In contrast, although no distinct trend is observed during diastole, the time‐averaged shear force is 0.0410 for torsion ON, which is 4.87% lower than that of torsion OFF.

**FIGURE 6:**
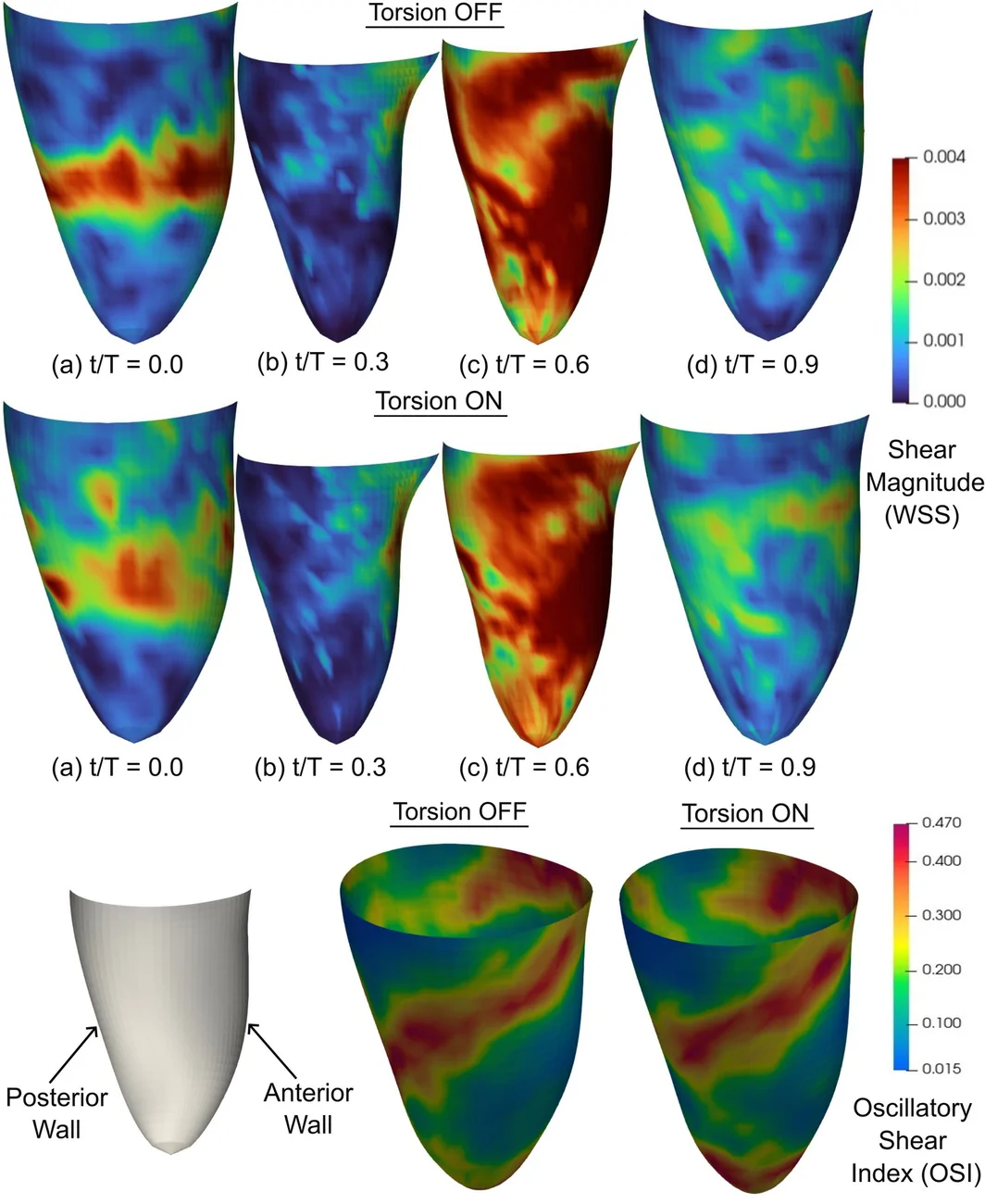
Nondimensional WSS magnitude contours on the LV surface split along the A3C view (row 1 and row 2) and 3D view of OSI contours (row 3) on the reconstructed LV chamber for Torsion ON and Torsion OFF cases. WSS is visualized at several cardiac instants t/T: (a) early systole; (b) mid systole; (c) early diastole; and (d) mid diastole.

Oscillatory Shear Index (OSI) is useful for characterizing directional changes and fluctuations in the magnitude of WSS throughout the cardiac cycle [56]. OSI ranges from a minimum of 0 (purely unidirectional flow) to a theoretical maximum of 0.5, which corresponds to flows with strong WSS oscillations. The LV OSI contour in Figure 6 shows a predominant region of noncyclic flow behavior and a spiral‐like band of elevated WSS oscillations in both torsion OFF and ON configurations. OSI values are observed to be higher in the torsion ON case near the apical region. Nonetheless, the area‐averaged OSI for the torsion ON case is recorded to be 0.199, which is 5.23% lower than the torsion OFF configuration.

The variation in the 3D flow separation and attachment patterns due to torsion is analyzed by studying the wall shear lines, also known as limiting streamlines [57]. Limiting streamlines are plotted as black lines while tangential LV surface velocity lines as red ones in Figure 7. For the Torsion ON case, the presence of slanted red streamlines serves as visual indicator for torsional motion. Flow separation along the surface normal direction is characterized by a stable node, where limiting streamlines converge, while flow attachment is indicated by an unstable node, where streamlines diverge. Both types of nodes are typically found in regions with very low WSS. At early systole (t/T=0), the Torsion ON case shows flow detachment near the anteroseptal wall, indicated by black lines extending from the apical region and converging at the basal section. In contrast, the Torsion OFF case exhibits flow attachment near the posterior wall without any notable detachment at the basal region. During diastolic filling (t/T=0.6), both cases reveal a stable spiral pattern in regions of minimal WSS, as illustrated in Figure 6.

**FIGURE 7:**
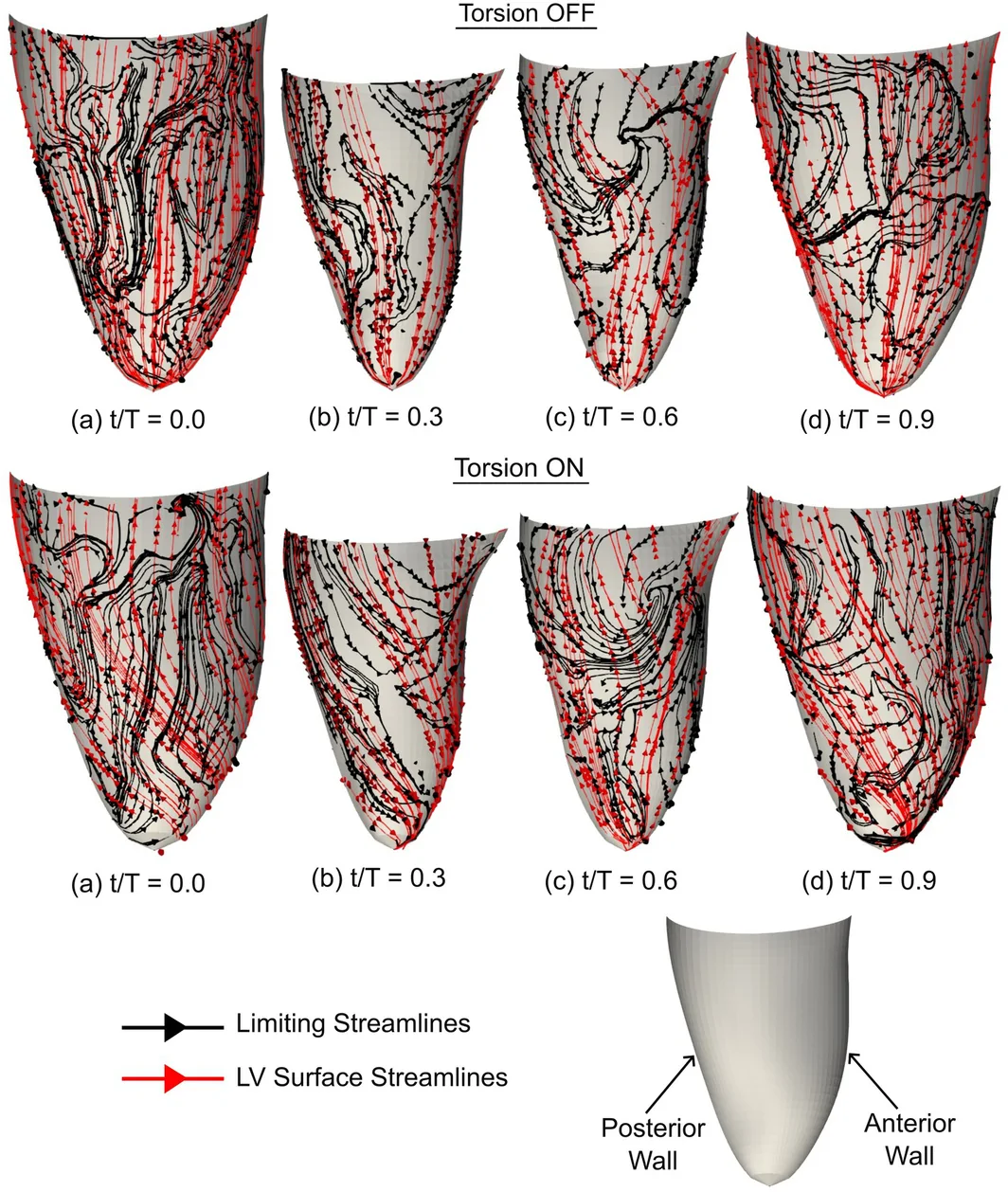
Limiting streamlines (black), showing the shear stress direction on the walls, and surface velocity streamlines (red) are plotted on the LV surface split along the A3C view at several cardiac instants t/T: (a) early systole; (b) mid systole; (c) early diastole; and (d) mid diastole.

Let us now shift focus from the fluid layers near the LV wall to the overall flow field and the residence time $\left(T_{R}\right)$. The scalar $T_{R}$ field is computed over the entire cardiac cycle by solving the transport equation using the known flow field and the boundary conditions described in Section 2.4. Based on the cycle‐to‐cycle sensitivity study for $T_{R}$ (Section 3.2), the results from the seventh cycle are discussed in what follows. The scalar field is normalized as $t_{R}=\frac{T_{R}}{T}$, where T is the cardiac cycle duration, and plotted as contours on mid‐plane along the A3C view in Figure 8. The same figure also shows the vorticity and velocity field contours. For analysis, locations with $T_{R}\leq T$ ($t_{R}\leq 1$) are considered to contain fresh blood, those with $T<T_{R}\leq 2T$ ($1<t_{R}\leq 2$) as aged blood, and regions where $T_{R}>2T$ ($t_{R}>2$) as stagnation zones. At the start of systole, low $t_{R}$ values appear within the LV due to A‐wave filling from the previous diastole. As systole progresses, $t_{R}$ increases locally due to the source term in the transport equation and the absence of new inflow. By ES, the LV is mostly filled with aged blood, along with isolated zones of stagnant fluid. With the onset of diastole, fresh blood ($t_{R}=0$) begins entering from the atrium. The formation and subsequent breakup of the diastolic vortex ring into smaller eddies enhances mixing, reducing $t_{R}$ within the LV.

**FIGURE 8:**
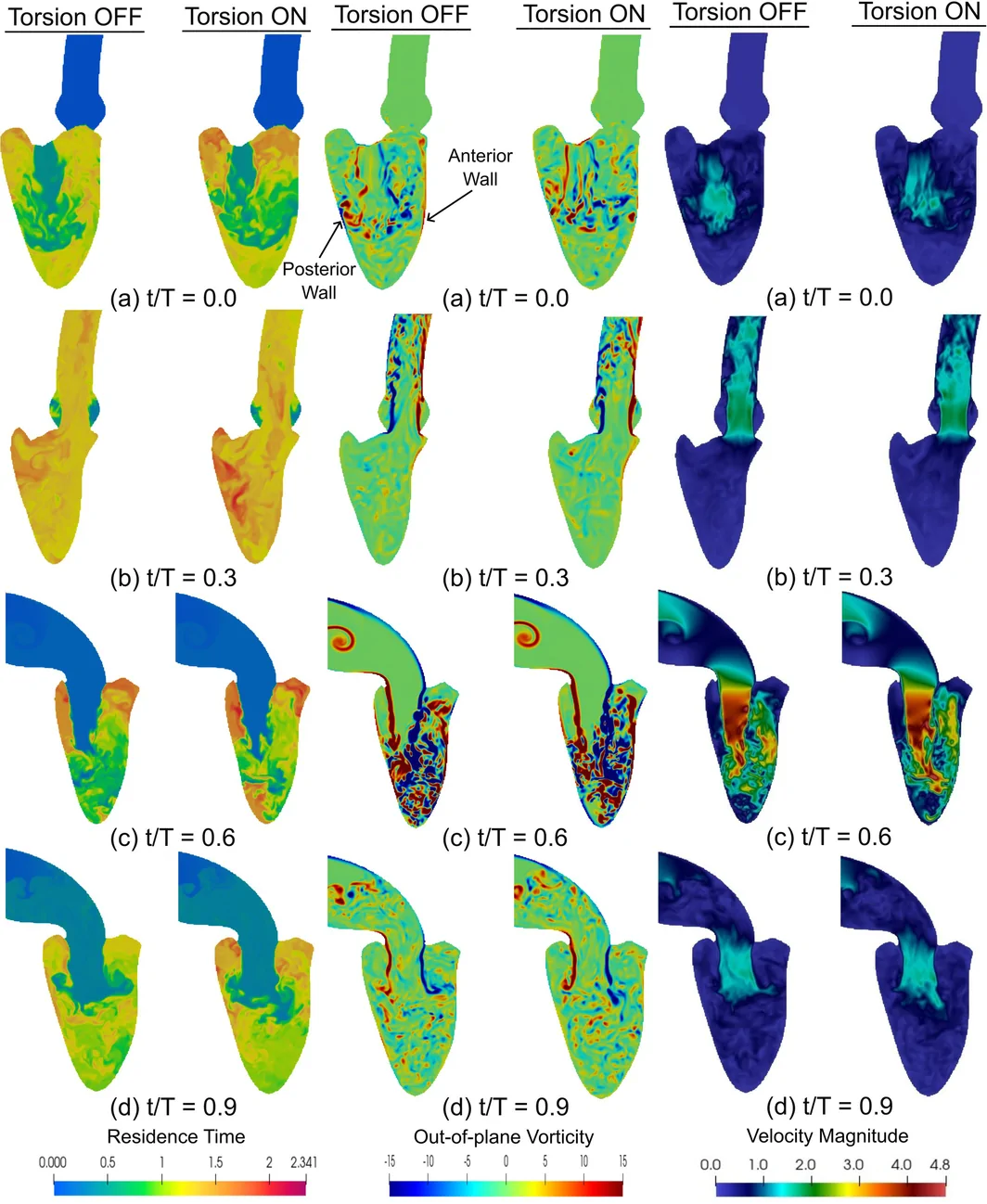
Comparison of nondimensional contours of residence time, vorticity, and velocity plotted on the mid‐plane along the A3C view at several cardiac instants t/T: (a) early systole; (b) mid systole; (c) early diastole; and (d) mid diastole.

For both torsion OFF and ON configurations, a region enclosed by the LV posterior wall, lid, and mitral valve (top‐left corner of the LV) consistently exhibits elevated $t_{R}$ throughout the cardiac cycle. In this same region, residence time is higher for the torsion ON case, indicating a persistent poor washout zone. Vorticity contours reveal that areas with higher vorticity within the LV promote stronger flow mixing, resulting in lower $t_{R}$ compared to adjacent regions. $t_{R}$ is also lower in regions experiencing higher diastolic inflow velocity. At t/T=0.6, two key observations can be made regarding the influence on residence time—one related to vorticity and the other to velocity. First, a portion of the apical region exhibits elevated residence time in the torsion ON case, which corresponds to a localized reduction in vorticity. Second, the tip of the diastolic jet is directed toward the anteroseptal wall for the torsion ON configuration, whereas it is oriented toward the posterior wall in the torsion OFF case. These jet tips also show lower $t_{R}$ values due to stronger inflow. Overall, in terms of residence time, torsion introduces specific regions with elevated $t_{R}$, while those same regions maintain relatively lower $t_{R}$ values throughout the cycle when torsion is absent.

The impact of torsion is better understood from the time history plot of volume fraction under different $T_{R}$ categories shown in Figure 9. Compared to the torsion OFF case, the torsion ON configuration shows a 16.59% reduction in the cycle‐averaged volume fraction of fresh fluid and a 3.7% increase in aged fluid, a trend also observed by Canè et al. [10]. Interestingly, in the torsion OFF case, the volume fraction of fresh fluid exceeds that of aged fluid during a few instances of diastole—a pattern not observed with torsion ON. The time history plot also shows that there is an insignificant amount of volume with stagnant fluid $\left(T_{R}>2T\right)$ without torsion. On the other hand, torsion ON case is recorded with stagnant fluid during late systole and early diastole. A maximum of $T_{R}=1.65\;s$ $\left(t_{R}=2.341\right)$ is recorded with 4.53% of total LV volume exhibiting residence time exceeding two cardiac cycles at ES.

**FIGURE 9:**
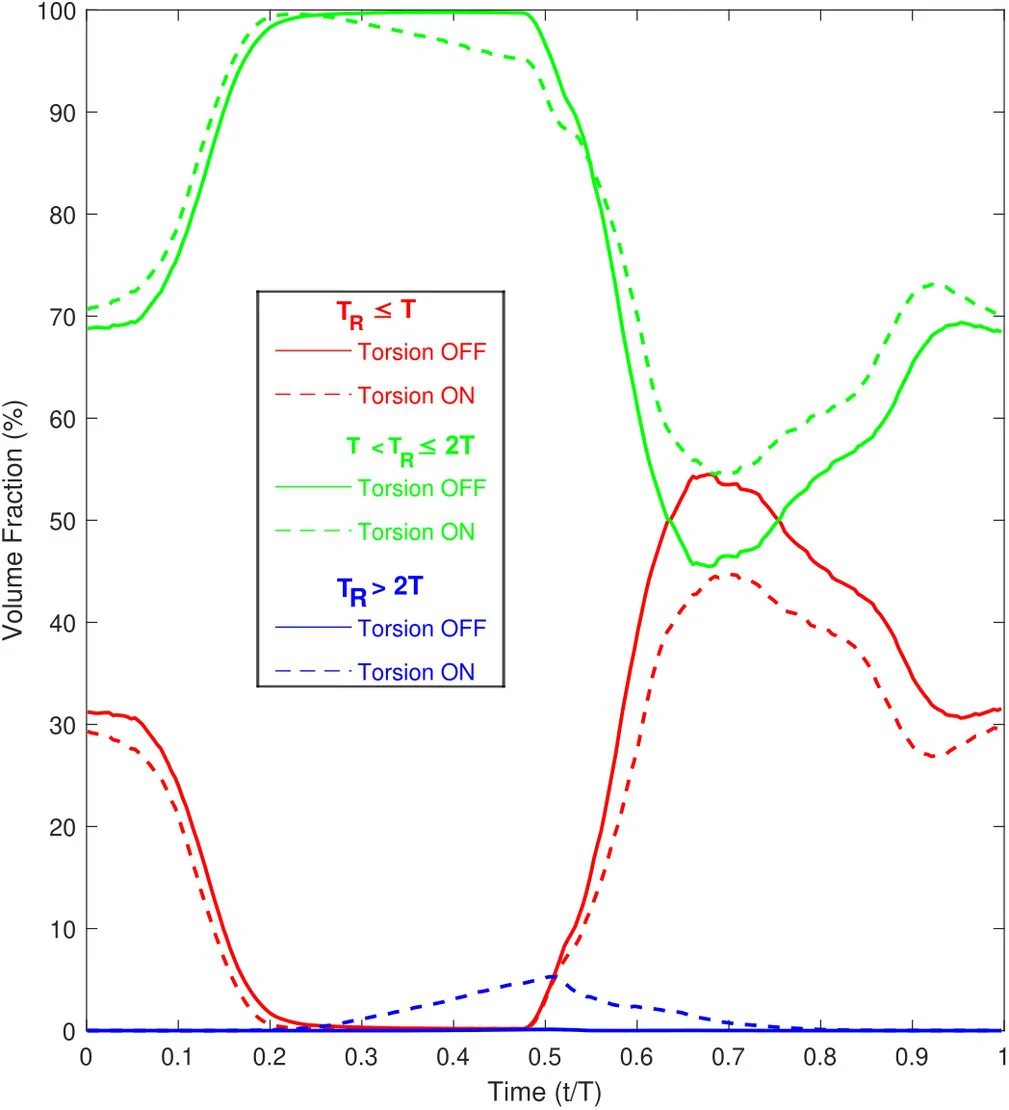
Time history of volume fraction for different categories of residence time. The colors denote the residence time, while the solid and dashed lines denote the torsion OFF and ON cases, respectively.

In summary, the addition of torsion in the 3D reconstruction of the LV from this set of images does not significantly alter the velocity fields, flow rate, or hemodynamic parameters such as SV when compared to the torsion OFF case. However, a noticeable reduction in $e_{\text{loss}}$ and an increase in the systole time‐averaged WSS are observed. Based on the residence time analysis, inclusion of torsion in LV reconstruction appears to reduce the LV's washout efficiency, as the percentage of fresh fluid $\left(T_{R}\leq T\right)$ is consistently lower, and that of aged fluid $\left(T<T_{R}\leq 2T\right)$ is higher across most time instances when compared to the simulation without torsion. Furthermore, torsion increases the residence time in certain regions, resulting in fluid persisting beyond two cardiac cycles, indicating stagnation zones in this subject.

## 4 Discussion

In the current study, torsion is introduced by rotating the short‐axis (SA) sections perpendicularly to the longitudinal direction of the left ventricle (LV), and its effect on hemodynamic parameters is analyzed. A maximum twist of 15° is observed in the LV due to torsional motion in the present open‐chest specimen. This maximum twist is comparable to the range reported for both open‐chest porcine (15.4°±5.10°) [58] and closed‐chest human LVs (9.3°–17.8°) [59, 60, 61]. This suggests that, while the open‐chest setup is a methodological limitation, the torsional rotation angles measured here are consistent with physiological twist magnitudes reported in both open‐ and closed‐chest settings.

Since the delineated SA sections are circular and the twist is of order 0.015° between two consecutive time steps, the torsional motion of SA sections generates tangential velocity components $\left(u_{t}\right)$ on the LV surface but does not significantly alter the shape of the SA sections. As a result, cardiac volumetric parameters did not exhibit significant changes with and without the inclusion of torsion in the 3D LV reconstruction from the same set of images. For pathological LVs with regional wall‐motion abnormalities (e.g., myocardial infarction with localized bulging), larger deviations from circularity of LV's SA sections may lead to non‐negligible changes in the reconstructed geometry from the same set of images when torsion is included. This should not be confused with structural simulations [15] or comparing diseased and healthy LV^18^ where torsion is reported to increase EF due to the helical arrangement of myocardial fibers. Here, torsion ON and OFF represent reconstruction with and without torsion from the same set of images of the same animal with a normal torsional motion, that is, it is not from two different animals with normal and abnormal LV torsion.

The time‐ and surface‐averaged wall shear stress (WSS) was reported to negligibly increase by 0.1% and $\left(e_{\text{loss}}\right)$ decrease by 0.5% due to torsion, as reported in an adult porcine study using a CFD‐MRI framework [62]. In the current study, similarly, WSS negligibly increases by 0.07%, and $e_{\text{loss}}$ decreases by 3.8%. In the MRI‐based study by Canè et al. [10], a 4% decrease in energy loss was reported based solely on the LV surface, excluding the left atrium, aorta, and valves. Our findings are consistent with this trend. The reduction in viscous energy loss under torsion can be attributed to improved flow organization and reduced oscillatory shear within the left ventricle. The area‐averaged OSI is 5.23% lower in the torsion ON case, indicating more unidirectional wall shear and fewer flow reversals. This suggests reduced shear layer interactions and diminished recirculatory losses, which in turn lowers viscous dissipation. Consequently, torsion promotes more efficient intraventricular transport, leading to reduced energy loss.

Canè et al. [10] also reported that torsion reduces the number of particles with residence time $\left(t_{R}\right)$ under one cycle by 7.9% and increases those with $t_{R}$ between one and two cycles by 3.0% for a left ventricular system without valves reconstructed from MRI images. Our results align with this trend, where the volume fraction of fresh blood $\left(T_{R}\leq T\right)$ decreases by 16.6% and aged blood $\left(T<T_{R}\leq 2T\right)$ increases by 3.7%, indicating reduced LV washout efficiency.

There are several limitations in the current work. 2D echocardiographic views are affected by image noise and artifacts, making it challenging to capture finer anatomical structures like trabeculae during LV reconstruction. This noise particularly hampers the ability to accurately track valve outlines using speckle tracking, often requiring manual intervention. The reconstruction of the valves is also constrained by the limited number of projections as only three LA views are used for the mitral and aortic valves, resulting in their simplifications. Uncertainties in the valve reconstruction and endocardium identification may affect local flow near the valves and walls, thereby affecting local wall shear stresses and OSI. The integral quantities are typically less sensitive to local variations. The left atrium and aorta currently serve as input/output to the ventricle and are not anatomically accurate. Anatomically realistic left atrium and aortic geometries reconstructed based on available echo projections as well as valves and quantifying uncertainty in the simulated quantities will be pursued as part of future work. Finally, only one specific LV is investigated in this work. To generalize the conclusions to a population, a cohort needs to be studied and statistically analyzed to show that the differences are significant.

## 5 Conclusion

In this study, we present a 3D reconstruction of the LV from standard (routinely recorded) 2D echocardiographic projections. Torsion implementation involves rotating the SA sections around the LV axis based on basal and apical rotation angles. Based on geometric verification using sonomicrometry, the shortest distance from the reconstructed LV surface to the coordinates of the implanted piezoelectric transducers is measured to be 3.36mm at ES and 5.37mm at ED. These values fall within the expected range of myocardial thickness and are consistent with reported surface errors between 3D echocardiography and cardiac MRI geometries [54]. The 3D reconstruction of the LV from 2D echo is combined with our in‐house CurvIB method, that is, echo‐CFD framework, to obtain the hemodynamics. Validation against pulsed wave Doppler ultrasound measurements shows good agreement between the simulated and experimental velocity time histories. This supports the main novelty of our work, that is, complex torsional motion can be modeled and its impact on hemodynamics can be studied from a 3D reconstructed LV geometry using an echo‐CFD framework.

The impact of including torsion in 3D reconstruction from echo images on LV hemodynamics is evaluated by comparing CFD results obtained with and without torsion in geometric reconstruction for a single porcine subject using the echo‐CFD framework. For this investigated specimen, including torsion does not affect the 3D reconstructed LV shape from 2D echo, and consequently the main geometrical parameters, for example, volumes, flow rate, and EF, due to the close‐to‐circular shape of the short‐axis cross‐section. Torsion mainly affects the LV surface velocity of the reconstructed shape. The time‐averaged wall shear force, calculated from WSS on the LV surface, increases by 12.6% during systole and decreases by 4.9% during diastole for the torsion‐enabled geometry. Both the OSI and energy loss are reduced by 5.23% and 3.8%, respectively, due to torsion. Residence time analysis for this specimen shows that torsion decreases the washout efficiency of the LV. The volume fraction of fresh fluid (residence time <1 cycle) is reduced by 16.6%, while fluid with residence time between 1 and 2 cycles increases by 3.7% on average over the cardiac cycle. Additionally, stagnant fluid with residence time exceeding 2 cycles is observed in the LV from late systole through early diastole. These findings are specific to the investigated porcine subject. To determine whether torsion consistently promotes reduced LV washout efficiency, future studies must include simulations across multiple specimens followed by detailed statistical analysis.

## Author Contributions

**S.S.C.S.:** investigation, methodology, software, formal analysis, validation, visualization, writing – original draft and editing; **H.G.:** investigation, methodology, formal analysis, visualization, writing – review and editing; **M.K.:** data curation, formal analysis, writing – review and editing; **S.S.A.:** methodology, software, writing – review and editing; **M.B.:** data curation, funding acquisition, project administration, supervision, resources, writing – review and editing; **I.B.:** conceptualization, funding acquisition, methodology, project administration, resources, software, supervision, funding acquisition, project administration, writing – review and editing.

## Funding

This work was supported by the National Science Foundation (2152869).

## Ethics Statement

The animal study was approved by the Institutional Animal Care and Use Committee at Mayo Clinic.

## Conflicts of Interest

The authors declare no conflicts of interest.

## Data Availability

The data that support the findings of this study are available from the corresponding author upon reasonable request.
